# Impact of a multidisciplinary pain program for the management of chronic low back pain in patients undergoing spine surgery and primary total hip replacement: a retrospective cohort study

**DOI:** 10.1186/s13037-014-0034-5

**Published:** 2014-08-08

**Authors:** Nicolas H von der Hoeh, Anna Voelker, Jens Gulow, Ute Uhle, Rene Przkora, Christoph-Eckhard Heyde

**Affiliations:** 1 Department of Orthopedic Surgery, University Hospital Leipzig, Liebigstrasse 20, Leipzig, 04103, Germany; 2 Department of Psychology and Psychosomatic Medicine, University Hospital Leipzig, Semmelweissstr. 10, Leipzig, 04103, Germany; 3 Department of Anesthesiology, The University of Texas Medical Branch, 301 University Boulevard, Galveston, 77555, TX, USA

**Keywords:** Chronic low back pain, Surgery, Total hip replacement, Multidisciplinary pain program

## Abstract

**Background:**

Low back pain is a very common disorder. In this field chronic low back pain represents a special challenge. The management of chronic low back pain consists of a range of different intervention strategies. Usually operative intervention should be avoided if possible. However, there are constellations were surgical therapy in patients with chronic low back pain seems to be meaningful.

The aim of this study was to investigate the clinical outcomes after spine surgery and hip replacement in patients with chronic low back pain after undergoing a structured rehabilitation program including cognitive – behavioral therapy.

**Methods:**

From January 1, 2007 to January 1, 2010 patients were indicated for total hip replacement (THA) or spine surgery after receiving inpatient multidisciplinary pain programs including cognitive – behavioral therapy at our orthopedic institute with a specialized unit for the rehabilitation of chronic pain patients. Indications for surgery were based on the synopsis of clinical and imaging findings and on positive effects after local injections during the multidisciplinary pain program. The tools for assessment included follow-up at 6 and 12 months and analyses of pain, chronicity, physical functioning and depression.

**Results:**

Of the 256 patients admitted for multidisciplinary pain program, fifteen were indicated to benefit from a surgical intervention during multidisciplinary pain program. Ten patients received spine surgery. THA was indicated in five patients. In all cases, the peri- and postoperative clinical courses were uneventful. Only two of the patients subjected to spine surgery and three patients who had THA were improved after 12 months. One patient reported a worsened condition. All patients presented with good functional outcomes and normal radiological findings.

**Conclusions:**

The indication for surgical intervention in patients with chronic low back pain and degenerative diseases must be critically assessed. THA in this cohort should focus on functional aspects, such as the improvement of range of motion, rather than the reduction of pain. Spine surgery in chronic low back pain patients after multidisciplinary pain program including cognitive – behavioral therapy cannot be recommended due to its questionable success.

## Background

Low back pain is one of the most common physical disorders in industrialized countries. Approximately 10 percent of these patients present with a chronic course with varying degrees of severity [1],[2]. To date, chronic low back pain is one of the most common causes of long-term disability, which is reflected in a high and progressive socioeconomic burden worldwide [3]. With the help of advanced diagnostic modalities, the differentiation of the various causes underlying low back pain have significantly improved, allowing the pathological morphology to be localized in up to 75% of cases [4],[5]. Therefore, patients with chronic low back pain constitute an increasing challenge for every professional who treats spinal disorders. The management of chronic low back pain consists of a range of different intervention strategies, including physical therapy, occupational therapy, surgery, and pharmacological treatment, along with adjunctive treatment modalities, such as epidural steroid injections, acupuncture, and other alterative medical therapies [6]–[8].

In 1977, Engel [9] introduced the bio-psycho-social model of health and demonstrated the complexity of the development of chronicity. Hence, the focus on how to approach chronic low back pain changed from the observation of its structural progression towards an understanding of its multifactorial influences. The aim of chronic pain treatment has evolved from focusing on the elimination of pain to managing pain to an extent that the patient’s physical and emotional functioning and overall quality of life are improved. Multidisciplinary pain programs, including cognitive – behavioral therapy, seem to be more effective in reducing pain intensity than active treatments (e.g., exercise therapy, physical therapies) alone [10]. An multidisciplinary pain program staff needs to work together as a team to address both behavioral and emotional sequelae from longstanding pain that stand in the way of a successful outcome.

## Methods

We retrospectively analyzed all patients included in a prospective data bank from January 1, 2007 to January 1, 2010 with chronic non-specific low back pain who received inpatient treatment for multidisciplinary pain program and surgery for degenerative conditions of the lumbar spine or THA in our orthopedic institution. All were patients of our multidisciplinary unit that specializes in the rehabilitation of chronic low back pain and mixed pain from coexisting degenerative hip symptoms. Further assessment of the spine or hip pathology was achieved through careful observation of medical histories, physical examination findings, and all imaging studies [4],[11]. The latter included computerized tomography (CT), magnetic resonance imaging (MRI), radiographs of the spine, and pelvis, hip, or functional x-ray. Based on a multidisciplinary approach, every patient receiving multidisciplinary pain program was assessed by every speciality participating in the multidisciplinary pain program. Staffing in our multidisciplinary pain program includes at least one specialized pain therapist, a physical therapist, a psychologist and an orthopedic surgeon. The individual therapeutic goals were determined in grand rounds prior to the multidisciplinary pain program. An individualized treatment plan was received for every participating patient. All patients received intensive active exercise therapy to improve joint and muscle function. Also the patients were introduced and supervised to learn an individual do-it-yourself- exercise-program. Additionally, a psychologist provided health education, stress management training, and relaxation techniques (progressive muscle relaxation) to each patient as an integral part of the cognitive - behavioral therapy. Pharmacologic treatment, such as nonsteroidal anti-inflammatory drugs, acetaminophen, topical agents, antidepressants (including serotonin/norepinephrine reuptake inhibitors and tricyclic antidepressants), anticonvulsants and opioids, was adjusted by a specialized pain therapist. In addition, all included patients received specific injections to the lumbar or thoracolumbar spine or, in cases of morphologically evident arthritis, hip joint infiltration, which were administered by one of three orthopedic surgeons in this team. Theses injections included epidural steroid injections (interlaminar or transforaminal), injections to the facet joints, selective nerve root injections and hip joint injections. The professionals on our team were specifically trained in the care of chronic pain patients. We performed only one injection every day and documented the effects of the pain relief on the next day to achieve pain relief and to localize the major source of pain.

Patients with both at least 50 percent pain relief on an 11-point numeric rating scale (NRS) from the local anesthetic injections of the lumbar spine or hip and concordant clinical and radiological findings explaining the symptoms were indicated for surgical intervention and enrolled in our study. Those who benefited from the hip injections underwent a total hip replacement (THA), and those for whom the lumbar region injections were successful underwent spine surgery e.g. pain release after facet joint injection L4/5 and selective nerve root injections L5 correlating to clinical and radiological findings TLIF L4/5 was performed.

The clinical and radiological follow-up was assessed 6 and 12 months postoperatively. All detailed medical histories were recorded by the same professional who conducted the initial pain therapy. Data assessments of every patient were completed before multidisciplinary pain program, preoperatively and at each follow-up. Clinical outcome measures were evaluated with the 11-point pain intensity numerical rating scale (NRS) [12] based a pain assessment protocol, the chronicity stages from the Mainz Pain Staging System (MPSS) and the functional disability from the Hannover Functional Ability Questionnaire (FFbH-R) [13]. Depression was measured by the self-reported Patient Health Questionnaire (PHQ-9) [14].

## Results

In fifteen of the 256 enrolled patients, the clinical course and diagnostic evidence indicated a surgical intervention. These fifteen patients (n = 12 female, n = 3 male) with a mean age of 64 years (range 38–82 years) were eventually subjected to surgery. All patients had an average pain history of 10 months (range 6–17 months).

**Figure 1:**
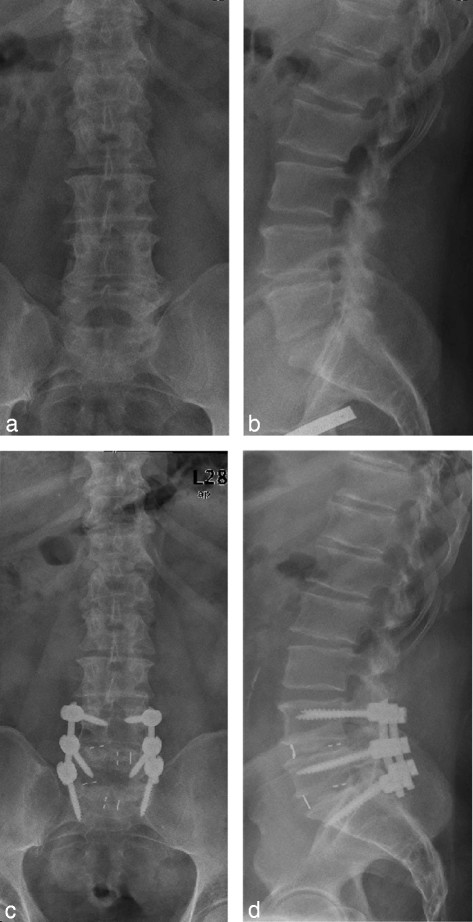
**A 56-year-old man with degenerative disease at levels L4/5 and L5/S1.** He benefited after probatory infiltration. One year after surgical treatment with two-level decompression and TLIF L4/5 and L5/S1, there were no changes in the measured score levels of chronicity, depression, activities of daily living and pain intensity compared to the preoperative/pre multidisciplinary pain program treatment scores. **a, b)** Preoperative x-ray, a.p. and lateral view. **c, d)** One year after surgery.

Ten patients underwent surgery on the lumbar or thoracolumbar spine (Table 1); their conditions included degenerative scoliosis, consecutive spinal stenosis of the thoracolumbar spine (n = 3) (Figure 1) and degenerative spondylolisthesis with consecutive spinal stenosis of the lower lumbar spine (n = 3). The surgical strategy consisted of interlaminar decompression, resection of the degenerated intervertebral disc and intervertebral fusion using a TLIF (transforaminal lumbar interbody fusion) approach with the implantation of a titanium cage with autologous bone grafting, completed by posterior pedicle screw-rod instrumentation. The three patients with lumbar spinal stenosis underwent microscopic bilateral interlaminar decompression in an undercutting technique that preserved the respective facet joints. One patient (n = 1) required posterior reinstrumentation due to a degeneration of the cranial adjacent segment two years after the lumbar fusion was performed.

**Table 1:** Detailed summary of the measured parameters in the spine surgery group (n = 10)

**Patient no.**	**1**	**2**	**3**	**4**	**5**	**6**	**7**	**8**	**9**	**10**
MPSS pre-op	3	3	2	3	2	2	2	2	2	2
MPSS 6-month-fu	3	3	2	3	2	2	2	2	2	2
MPSS 12-month-fu	3	3	2	3	2	2	2	2	2	2
PHQ pre-op	19	21	17	20	13	9	12	7	9	12
PHQ 6-month-fu	17	20	16	20	24	9	12	8	8	13
PHQ 12-month-fu	16	21	15	21	12	9	11	6	5	14
NRS pre-op	8.2	7	7.5	6	7.5	7.6	8.1	7.5	7	6
NRS 6-month-fu	6.6	5.4	6	2	7.5	4.3	5.4	3	2.5	9
NRS 12-month-fu	7	6.6	6.1	5	7.7	6.4	7.2	3.6	2.7	10
FFbH pre-op	54	55	50	58	56	63	63	70	67	53
FFbH 6-month-fu	60	55	56	58	52	63	50	70	79	43
FFbH 12-month-fu	58	37	50	54	55	62	45	73	75	47

Legends: Outcome scales: MPSS (Mainz Pain Staging System) for indicating chronicity stages, NRS (numerical rating scale) for measuring pain intensity, FFbH (Hannover Functional Ability Questionnaire) for indicating activities of daily living, and PHQ-9 (self-reported Patient Health Questionnaire) for depression.

Total hip replacement was indicated and performed in five patients (n = 5) with osteoarthritis of the hip joint accompanying the chronic back pain (Table 2). All patients experienced uneventful peri- and postoperative courses of recovery without complications.

**Table 2:** Detailed summary of the measured parameters in the THR group (n = 5)

**Patient no.**	**1**	**2**	**3**	**4**	**5**
MPSS pre-op	2	3	2	2	3
MPSS 6-month-fu	2	3	2	2	3
MPSS 12-month-fu	2	3	2	2	3
PHQ pre-op	25	20	12	13	15
PHQ 6-month-fu	22	22	5	7	11
PHQ 12-month-fu	28	21	5	9	12
NRS pre-op	9	6.8	6.9	6.2	7
NRS 6-month-fu	4	4.2	4.5	2.3	3
NRS 12-month-fu	7.5	6.2	3.1	3.3	1
FFbH pre-op	72	67	83	64	55
FFbH 6-month-fu	70	67	83	73	63
FFbH 12-month-fu	74	64	88	86	75

Legends: Outcome scales: MPSS (Mainz Pain Staging System) for indicating chronicity stages, NRS (numerical rating scale) for measuring pain intensity, FFbH (Hannover Functional Ability Questionnaire) for indicating activities of daily living, and PHQ-9 (self-reported Patient Health Questionnaire) for depression.

Four patients from the spine group (n = 3 after TLIF, n = 1 after decompression) reported an improvement of their pain symptoms at the 6-month postoperative follow-up. However, after 12 months, only two patients (n = 1 after TLIF and n = 1 after decompression) maintained their improved postoperative status. Worsening of pain was noted in one case (n = 1) after TLIF at the 6- and 12-month follow-up visits. In contrast, all patients presented good functional results and normal imaging findings in follow-up. All five patients receiving THA (n = 5) experienced improvements with reduced pain at 6 months postoperatively. However, only 3 patients (n = 3) maintained their pain improvement after 12 months postoperatively, again with unremarkable radiological findings and improved function.

The pain levels were unchanged in the remaining patients, without worsening or improvement. Additionally, the preoperative capacities to perform daily activities related to their disability within the spine-group were not significantly different compared to the postoperative values from the Hannover Functional Ability Questionnaire (HFAQ). In contrast, only two patients in the THA group (n = 2) showed improvement in both their pain and their HFAQ scores, while one patient (n = 1) presented decreased functional capacity with an increase of pain symptoms in the follow-up.

The psychologist’s reports confirmed persistent chronicity in all patients (100% of cases). During the observation period, MPSS levels remained consistent. Level II and III scores were identified in ten and five patients, respectively. The results of the PHQ-9 revealed evidence of mild depression in three patients (n = 3), moderate depression in 6 patients (n = 6) and moderately severe depression in two patients (n = 2). Four patients (n = 4) presented with severe depression. Two patients of the THA group improved after surgery, with changes from moderate to mild depression. In all other cases, there were no changes after 6 or 12 months in the PHQ-9 score values. All patients continued their daily intake of analgesic medications for ongoing pain syndromes. None of the patients were able to reduce their preoperative pain medications, such as non-steroidal anti-inflammatory drugs (NSAIDs), opiates or benzodiazepines, at any point throughout the follow-up period.

## Conclusions

Multidisciplinary pain programs that focus on physical, psychological, social and related factors have clear therapeutic aims and strategies. The objectives of multidisciplinary pain programs are the mild or moderate reduction of pain intensity and improvement in quality of life. Thus, the main goal of this rehabilitation program is functional restoration [5],[8],[15]. In the elderly population, osteoarthritis remains the most common musculoskeletal disease [11]. Thoracolumbar degenerative diseases that cause radiculopathy and claudication symptoms, in combination with overlapping pain from hip joint arthritis, can lead to a so-called “hip-spine syndrome”. Injections and diagnostic tests can reduce symptoms and are recommended to vary specifically by the concomitant locations of pain [16]. Multidisciplinary pain programs were not developed to serve as diagnostic tools for surgical intervention, particularly for patients suffering from chronic low back or mixed pain. Patients with chronic pain are prone to a progression of degenerative diseases that can maintain or increase the level of chronicity while influencing mobility and quality of life. In contrast to the common lack of response observed in chronic low back pain patients undergoing multidisciplinary pain program, our study initially demonstrated a clear relief of pain in 15 patients, with a recorded reduction of more than 50 percent after the injection of anesthetics. The decision to operate was made after interdisciplinary consultation with a focus on both successful injection and an underlying distinct degenerative pathology concordant with the clinical symptoms and pain relief after the successful injection. Farrar et al. demonstrated that, on an 11-point pain intensity numerical rating scale, a reduction of 1.8 points is equivalent to a change in pain of approximately 30%. This effect is considered a satisfactory result. An improvement in the NRS of 3 or more is equivalent to a change in pain of 50%, an extremely satisfying result for this patient cohort [12].

None of the patients demonstrated associated complications after surgical intervention. Nevertheless, only two patients from the spine group improved in their overall values. One patient even reported a worsened pain level. Eight patients in this group maintained their levels throughout the 12-month follow-up period. Despite proper functional and unremarkable radiological results, the presence of behavioral disorders or psychiatric comorbidities is well known to significantly impair the subjective clinical course in patients with musculoskeletal interventions [17]. Ibrahim et al. reported a marginal improvement in the Oswestry Disability Index in patients with chronic low back pain and spinal fusion compared to non-surgical interventions, but the difference was not statistically significant. Fusion surgery was found to be associated with a significant risk of complications [15]. Mirza et al. summarized that fusion may not be more effective than a structured rehabilitation program that includes cognitive - behavioral therapy [18].

In the THA group, three patients exhibited a reduction in pain symptoms without a change in the level of chronicity. In recent studies, up to 12.1% of patients undergoing THA develop chronic low back pain [19]. Regarding the FFbH-R results, there was evidence in the THA group of a correlation between disability (reduced functional capacity) and pain intensity, although disability was not associated with the duration of low back pain [20].

Depressive symptoms are very common in chronic pain patients. Studies have reported prevalences of 30-80% of patients with some depressive symptoms and 20% of patients who fulfill the criteria for a true major depressive disorder [21]. Boos et al. demonstrated that patients with acute spinal disc symptoms but without neurological deficits were distinct from pain-free patients in their presentation of depressive and psychosocial distress symptoms. Moreover, depression had the highest predictive value for developing low back pain, regardless of any morphological correlation [22]. The persistence of chronic musculoskeletal pain in depressive patients correlated significantly with the number of pain sites. Depression, state anxiety and somatization disorders have also been reported to have a strong influence on peri- and postoperative outcomes. These disorders can affect the patient’s subjective awareness and pain control, thus increasing peri- and postoperative pain. [23]. There is increasing evidence that the fear of pain, along with the fear of hurt or harm, are major influences in the development of chronicity [24].

In patients with chronic low back pain, yellow flags indicating diverse psychosocial prognostic factors for the development of disability following the onset of musculoskeletal pain are often present. Additionally, depression and psychosomatic disorders are common in patients receiving multidisciplinary pain programs. Hence, with the existence of these risk factors, an operative intervention is not recommended due to the increased incidence of developing postoperative pain and chronicity [7],[9],[10],[25]–[27]. With regard to our observations, the steady levels of MPSS chronicity stages demonstrated the high psychosocial influence in both investigated groups. Recent studies have shown that patients with chronic low back pain can improve their quality of life after multidisciplinary pain program [9],[10]. Bentsen et al. recommended psychosocial interventions focusing on emotions and coping with pain in patients with chronic low back pain after spinal fusion to alleviate pain intensity and improve quality of life in the postoperative course [28]. Hence, the literature lacks studies that investigate to what extent the educational component, psychotherapy and behavioral - training may positively influence the postoperative outcome when multidisciplinary pain program is received preoperatively.

In our cohort, clinical and radiological findings were evident, and probatory infiltrations eliminated pain locally. Nevertheless, despite the functionally and radiologically unremarkable findings, the postoperative results were disappointing. If the decision is made for a surgical intervention, the patient should be adequately informed about the expected effectiveness and outcome. Moreover, the achievable reduction in pain did not always fulfill the expectations of the patient [29]. Subsequently, those patients will require intensive explanation regarding the high rate of pain resistance despite adequate surgical results and improved functional quality.

In conclusion, the indication to perform any surgical intervention in patients with chronic low back pain and degenerative diseases must be critically assessed. In his conclusion, Don et al. emphasizes the poor correlation between apparent degenerative changes and the clinical presentation of chronic low back pain, arguing against the expectation that surgically modifying the degenerative process will be highly effective in modifying pain and disability [8]. High-quality randomized controlled trials comparing non-operative treatments with surgical interventions are lacking. Chronic low back pain patients with depression and certain personality disorders should preferentially be treated nonoperatively, in consideration of red-flag conditions [30]. Our observations suggest that spine surgery in chronic low back pain patients after multidisciplinary pain program including cognitive – behavioral therapy cannot be recommended due to its questionable success. Total hip replacement in this cohort should focus on functional aspects, such as improvement of the range of motion, rather than on the reduction of pain intensity.

### Consent

Patients were informed verbal and in written form and confirmed their approval on a consent form.

## Competing interests

The authors declare that they have no competing interests.

## Authors’ contributions

NHVDH made substantial contributions for conception, design, analysis and interpretation of the data. AV did the main part of data acquisition. JG has been involved in drafting the manuscript. RP have been involved in design and analysis of the data. UU has been involved in analyses of the psychological data. CEH has given final approval of the version to be published. All authors read and approved the final manuscript.
